# Neural Process of the Preference Cross-category Transfer Effect: Evidence from an Event-related Potential Study

**DOI:** 10.1038/s41598-017-02795-w

**Published:** 2017-06-09

**Authors:** Qingguo Ma, Linanzi Zhang, Guanxiong Pei, H’meidatt Abdeljelil

**Affiliations:** 1 Institute of Neuromanagement Science, Zhejiang University of Technology, Hangzhou, China; 2 School of Management, Zhejiang University, Hangzhou, China; 3 Neuromanagement Lab, Zhejiang University, Hangzhou, China; 4 School of Management, Guizhou University, Guiyang, China

## Abstract

In business practice, companies prefer to find highly attractive commercial spokesmen to represent and promote their products and brands. This study mainly focused on the investigation of whether female facial attractiveness influenced the preference attitudes of male subjects toward a no-named and unfamiliar logo and determined the underlying reasons via neuroscientific methods. We designed two ERP (event-related potential) experiments. Experiment 1 comprised a formal experiment with facial stimuli. The purpose of experiment 2 was to confirm whether the logos that were used did not present a significant difference for the subjects. According to the behavioural results of experiment 1, when other conditions were not significantly different, the preference degree of the logos correlated with attractive female faces was increased compared with the logos correlated with unattractive faces. Reasons to explain these behavioural phenomena were identified via ERP measures, and preference cross-category transfer mainly caused the results. Additionally, the preference developed associated with emotion. This study is the first to report a novel concept referred to as the “Preference Cross-Category Transfer Effect”. Moreover, a three-phase neural process of the face evaluation subsequently explained how the cross-category transfer of preference occurred and influenced subject preference attitude toward brand logos.

## Introduction

In business practice, companies prefer to find highly attractive commercial spokesmen to represent their products and brands. Most companies intended to use attractive men or women to promote their products and brands. In contrast, many online sellers on Taobao.com, authorized by Alibaba Group, have asked unattractive customers to withdraw their positive feedback show on the website. This issue created substantial interest to investigate the internal neural mechanism to explain this phenomenon. Did the attractive or unattractive individuals really impact consumer choices? According to our experimental results, the answer was “Yes”, and a reasonable explanation of this behaviour may be a result of preference cross-category transfer effect. Additionally, the preference developed associated with emotion.

Emotion is a rapidly changing psychological and physiological phenomenon, which reflects the body’s adaptation to the changing environment. In recent years, a substantial number of studies have investigated emotional perception and its brain mechanism. Neural imaging studies have demonstrated the separation of positive emotion and negative emotion^1, 2^. Using fMRI techniques, a significant increase in the signal intensity in the left amygdala was identified during the induction of a sad mood; however, there was no comparable effect during the induction of a happy mood. A subset of these studies adopted another neuroscience measurement, Event-Related Potentials (ERPs), and focused on the neural mechanism of how affective pictures induce emotions in individuals^3–8^. Olofsson *et al*.^9^ performed an integrative review of the ERP findings of affective picture processing in 2008. According to their findings, differences in components were identified when different moods were induced. Lifshitz^10^ demonstrated that pleasant and unpleasant pictures induced a positive-going waveform at approximately 350–450 ms after stimulus onset compared with neutral pictures from the 1960s of the 20^th^ century. Schupp *et al*.^11^ also identified larger Late Positive Potentials (LPP) when subjects viewed pleasant and unpleasant pictures compared with neutral pictures. Researchers have also utilized facial expression pictures (happy and angry faces) as stimuli, and early brain electrical components (e.g., N170 and P1) were observed during the experiments^12–14^. Other studies have indicated that Late Positive Components (LPC, e.g., P300 and LPP) were produced when affective facial pictures were presented^15–19^.

Studies using ERP experiments which focused on facial attractiveness usually reported EPN (early posterior negativity) and LPC (late positive component) to explain the neural process of subjects. Johnston and Oliver-Rodriguez^20^ first used ERP to investigate the cognitive processing of the brain to attractive human faces. The results indicated the amplitudes of LPP correlated with the differential responses of facial attractiveness. Furthermore, different levels of attractiveness caused the difference in the LPP in the time-window of 400–600 ms, and an early posterior negativity (EPN) during 230–280 ms was also identified in the frontal region of the brain^21^. Although a contrary LPP pattern has been identified^22^, an early negativity (N2) and a similarly late LPP component were investigated in the same two temporal stages of processing attractive and unattractive faces. Chen *et al*.^22^ reported that a smaller P2 amplitude was elicited by attractive faces compared to unattractive faces and attractive faces elicited larger N2 and smaller late positive component (LPC) amplitudes than unattractive faces. Munoz and Martin-Loeches^23^ observed an increased P300 when beautiful images presented compared to ugly ones. Sun *et al*.^24^ investigated facial expressions and reported the components P2l and P2m, which correlated with facial attractiveness and facial expression, and the component LPP, which correlated with attention. In the early process, P2l and P2m were processed separately for discrimination between stimuli during the early stage of face perception. In later processing, LPP “would be allocated to the faces with the most positive or most negative valences in either attractiveness or expression”. Academicians have also been interested in female facial attractiveness with respect to its strong impact on decision making in social lives. Previous studies have demonstrated that a “beauty premium” existed in social societies, which indicated an attractive appearance in life and social communication may lead more benefits, such as job opportunities and chances, compared with unattractive individuals^25–33^. Furthermore, facial attractiveness influenced the fairness consideration of individuals during social interactions through the Ultimatum Game^34, 35^. There are many other documents regarding the neural processes of facial attractiveness. We only selected representative papers to discuss.

This study adopted ERPs to record brain activities, with an experimental paradigm that used female attractive or unattractive faces without expression as stimuli and a simple task for subjects to provide their preference to a fabricated logo on the screen to investigate how facial attractiveness influences subject preference. Through the experimental results, we demonstrated how the attractive (or unattractive) female faces induced different preference degree associated with positive (or neutral) emotions in the subjects and how the preference was transferred across categories (from human faces to brand logos) ultimately.

## Results

### Behavioural Results

#### Results for Experiment 1

A paired-samples T test was used to analyse the rank scores of the preference degree on the logos and facial attractiveness ratings. According to the results, the subjects ranked a higher score to the logos matched to the attractive female recommenders ($Mea{n}_{Logo-Attractive}$ = 3.438, SD = 0.511; $Mea{n}_{Logo-Unattractive}$ = 2.3, SD = 0.725), and the T test was significant (t = 7.82, p < 0.01). The T test was also significant of the facial attraction rating ($Mea{n}_{Attractive}$ = 3.401, SD = 0.43; $Mea{n}_{Unattractive}$ = 1.803, SD = 0.48, t = 24.699, p < 0.01). Furthermore, the results of a bivariate correlations between attractiveness rating scores and logo preference degrees showed that the valence score of facial attractiveness was positively correlated with the logo preference degree ($Pearso{n}_{Attractive-Logo}$ = 0.608, p = 0.01; $Pearso{n}_{Unattractive-Logo}$ = 0.497, p = 0.042).

#### Results for Experiment 2

A paired-samples T test was also used to analyse the rank scores of the preference degree for the logos in this experiment. Without face stimuli, the subjects ranked almost no differences in scores between the logos matched to the attractive and unattractive female recommenders in experiment 1 ($Mea{n}_{Logo-Attractive\text{'}}$ = 2.531, SD = 0.527; $Mea{n}_{Logo-Unattractive\text{'}}$ = 2.577, SD = 0.599), and the T test was not significant (t = 0.468, p = 0.646).

### ERP Results

#### Experiment 1

**Figure 1:**
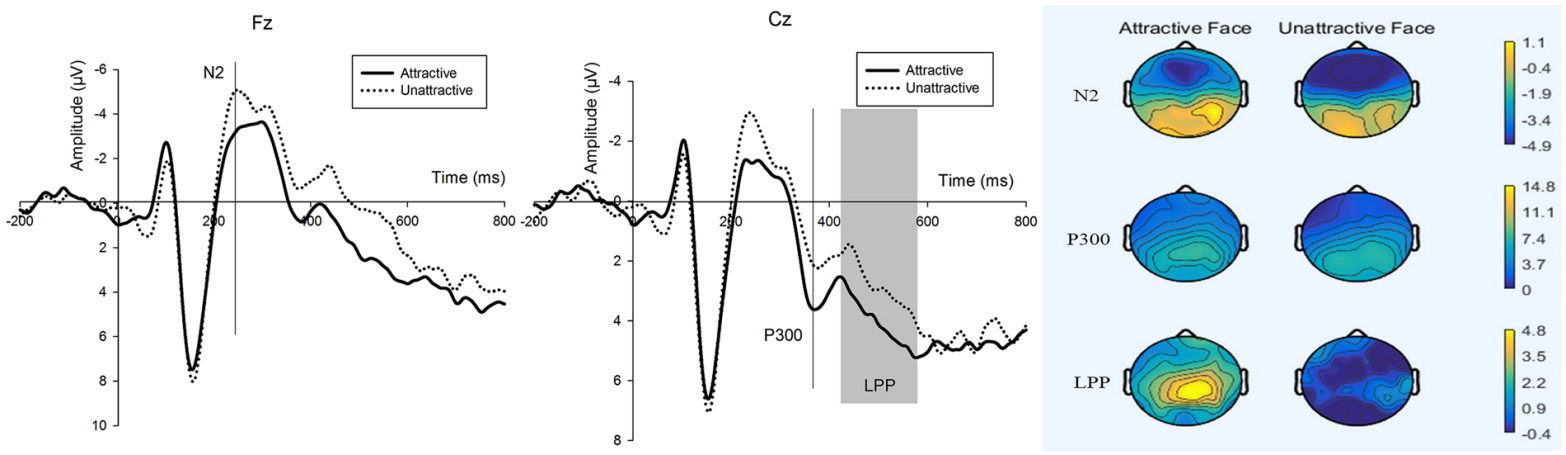
Wavefroms and brain topographic maps of N2, P300 and LPP of female faces onset in experiment 1. Fz and Cz were chosen to represent.

Face Onset (see Fig. 1) N2 the main effect of face attractiveness was significant (${F}_{1,18}$ = 6.439, p = 0.021), and the mean potential peak amplitude value of the six electrodes (F1, Fz, F2, FC1, FCz, FC2) was −4.449 μV ($Mea{n}_{N2-Attractive}$ = −4.449 μV, SD = 4.188); when the subjects viewed unattractive faces, a more negative N2 component was identified ($Mea{n}_{N2-Unattractive}$ = −5.897 μV, SD = 5.191). The effect of the electrodes (*F*_1,18_ = 2.207, p = 0.061) and the interaction effect between the face stimuli and electrodes (*F*_1,18_ = 0.764, p = 0.579) were not significant.

P300 according to the statistical analysis, the main effects of face attractiveness [$Mea{n}_{P300-Attractive}$ = 6.388 μV, SD = 4.965; $Mea{n}_{P300-Unattractive}$ = 4.971 μV, SD = 4.719 (the mean potential peak amplitude value of C1, Cz, C2, CP1, CPz and CP2); *F*_1,18_ = 10.103, p = 0.005] and electrode sites (*F*_1,18_ = 3.983, p = 0.021) were both significant. We identified a more positive P300 component with the onset of attractive female faces. The interaction effect between face and electrodes was not significant (*F*_1,18_ = 0.671, p = 0.652).

LPP the ANOVA analysis for the parietal LPP indicated a main effect of face, and a more positive LPP (*F*_1,18_ = 7.799, p = 0.012) was elicited by attractive faces (Fig. 3). The main effect of electrode (*F*_1,18_ = 11.843, p < 0.01) was also significant. However, the interaction effect between face and electrode was not significant (*F*_1,18_ = 0.875, p = 0.476).

**Figure 2:**
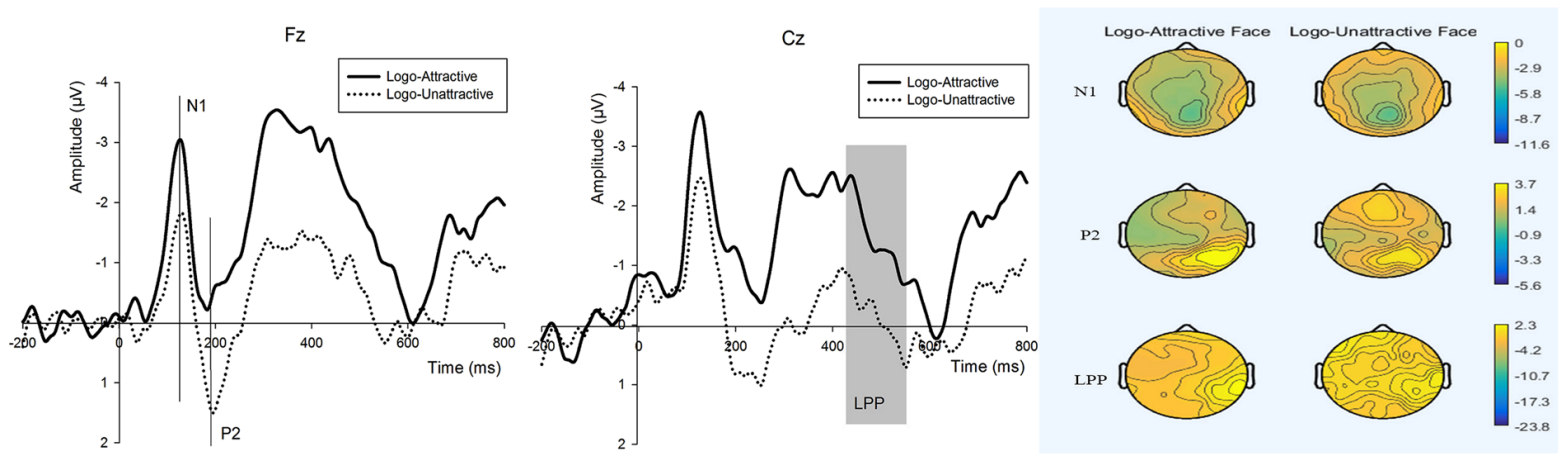
Wavefroms and brain topographic maps of N1, P2 and LPP of logo onset in experiment 1. Fz and Cz were chosen to represent.

Logo Onset (see Fig. 2) N1 the amplitude of the N1 component was significant [$Mea{n}_{N1-\mathrm{Logo}-Attractive1}$ = −4.341 μV, SD = 2.83; $Mea{n}_{N1-\mathrm{Logo}-Unattractive1}$ = −3.013 μV, SD = 2.113 (the mean potential peak amplitude value of F1, Fz, F2, FC1, FCz and FC2); *F*_1,18_ = 4.945, p = 0.04] between the two logo groups (logos correlated with attractive faces and logos correlated with unattractive faces); a more negative peak amplitude was identified in the group correlated with attractive faces. The main effect of the electrode (*F*_1,18_ = 2.682, p = 0.07) and the interaction effect between the logo and electrode were not significant (*F*_1,18_ = 0.642, p = 0.672).

P2 the ANOVA analysis for the early P2 component indicated main effects of logos followed by attractive faces, and a more positive P2 amplitude [$Mea{n}_{P2-\mathrm{Logo}-Attractive1}$ = 1.096 μV, SD = 3.309; $Mea{n}_{P2-\mathrm{Logo}-Unattractive1}$ = 2.689 μV, SD = 3.564 (the mean potential peak amplitude value of F1, Fz, F2, FC1, FCz and FC2); *F*_1,18_ = 8.841, p = 0.009] was elicited. However, the main effect of electrode (*F*_1,18_ = 0.665, p = 0.657) and the interaction effect between logo and electrode (*F*_1,18_ = 0.185, p = 0.963) were not significant.

LPP during the logo onset stage, the analysis of the LPP also indicated a main effect of logo; a more positive LPP (*F*_1,18_ = 6.614, p = 0.02) was elicited by logos matched with unattractive faces. The main effect of electrode (*F*_1,18_ = 1.432, p = 0.277) and the interaction effect between electrode and logo (*F*_1,18_ = 0.652, p = 0.666) were not significant.

#### Experiment 2

**Figure 3:**
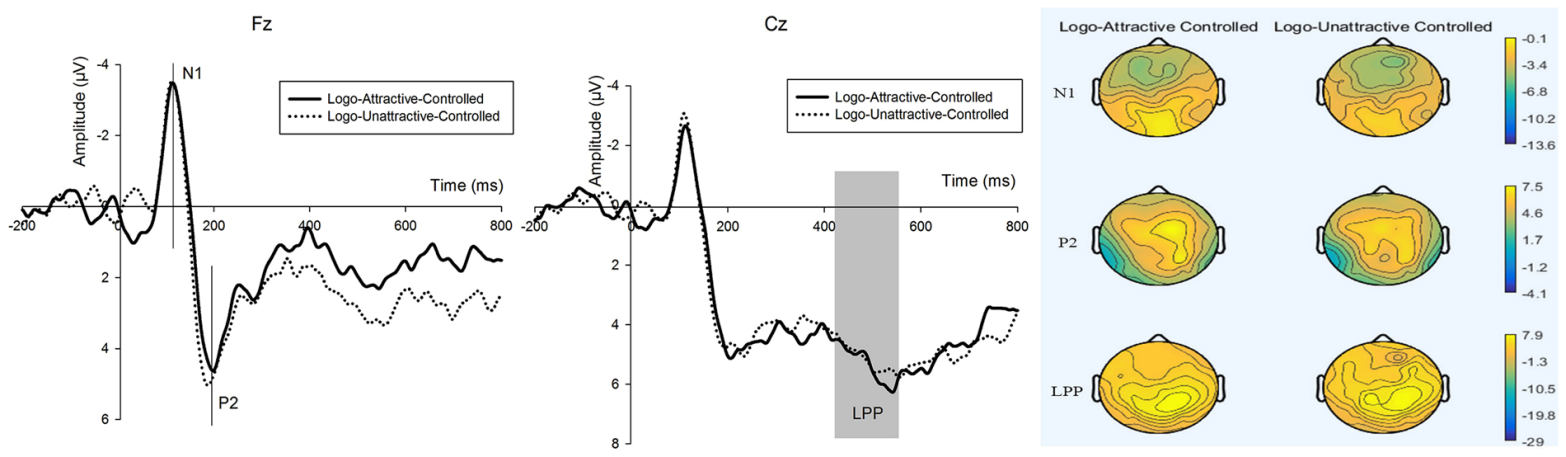
Wavefroms and brain topographic maps of N1, P2 and LPP of logo onset in experiment 2. Fz and Cz were chosen to represent.

Logo onset (see Fig. 3) the same time windows of the N1 and P2 components in the experiment group when the logo onset were selected to analyse the main effects in experiment 2. We used repeated measures ANOVA to determine whether the latency was different. The latencies of six electrodes from centrofrontal (F1, Fz, F2, FC1, FCz and FC2) were conducted in the analysis. The results indicated that there were no significant differences in the latency between the two situations. (${F}_{N1Latency-LogoAttractive}$ = 4.057, p = 0.061; ${F}_{N1Latency-LogoUnattractive}$ = 2.296, p = 0.148; ${F}_{P2Latency-LogoAttractive}$ = 2.322, p = 0.146; ${F}_{P2Latency-LogoUnattractive}$ = 0.011, p = 0.917).

N1 the amplitude of the N1 component was not significant ($Mea{n}_{N1-Logo-Attractive2}$ = −4.55 μV, SD = 3.197; $Mea{n}_{N1-Logo-Unattractive2}$ = −4.698 μV, SD = 3.0597; *F*_19,36_ = 0.198, p = 0.662) between the two logo groups (logos correlated with attractive faces and logos correlated with unattractive faces in the experiment group). The main effect of electrode (*F*_19,36_ = 2.134, p = 0.126) and the interaction effect between logo and electrode (*F*_19,36_ = 1.729, p = 0.197) were not significant.

P2 the ANOVA analysis for the early P2 component indicated no main effects of the two groups’ logos ($Mea{n}_{P2-Logo-Attractive2}$ = 6.031 μV, SD = 4.79; $Mea{n}_{P2-Logo-Unattractive2}$ = 5.7889 μV, SD = 5.632; *F*_19,36_ = 0.098, p = 0.758). The main effect of electrode (*F*_19,36_ = 3.21, p = 0.042) was significant and the interaction effect between logo and electrode (*F*_19,36_ = 2.038, p = 0.14) was not significant.

LPP the main effect of logo (*F*_19,36_ = 0.209, p = 0.653) and the interaction effect between electrode and logo (*F*_19,36_ = 1.528, p = 0.248) were not significant.

## Discussion

The main objective of this study was to analyze whether facial attractiveness would impact subjects’ preference attitude toward no-name and unfamiliar brand logos through neuroscientific measures. The results of behaviour and ERP measures of experiment 2 (Fig. 3) showed that the subjects’ attitudes regarding the selected logos were discrete and not significantly different when all influenced factors such as colours, patterns, and product properties were controlled. However, the rating scores of the same logos presented in experiment 1 were significantly different. In addition, ERP results demonstrated the differences as well.

The experimental paradigm we used was preference priming which might be different from affective priming as there were many reasons for the formation of preference and emotional changes was merely one of the causation. In our study, different preference attitude of female attractiveness was primed by different emotions according to the experimental results and those preferences transferred across categories to no-name logos. Finally, subjects’ behaviour was influenced by the “Preference Cross-Category Effect” which we proposed.

During the logo presentation period in experiment 1, two main stages of neural components were identified. N1 and P2 in early processing followed the LPP component in the later cognitive stage. The preference degree of the logos for which attractive female customers recommended was increased compared with the logos recommended by unattractive female customers. One potential explanation may be a result of the cross category transfer of preference effects (from physical attractiveness to unfamiliar logos). The ERP results also offered evidences from the neural process of the phenomena.

In the early cognitive stage when logo was onset in experiment 1, the N1 and P2 components were activated by observation. N1 has been considered a sensitive index of emotional valence in some studies, whereas other studies have demonstrated that positive or negative stimuli evoked comparable N1 components^36–41^. P2 has been considered a reflection of negativity bias; moreover, a larger P2 was elicited by negative pictures compared with positive and neutral pictures^42–46^. In our case, logos recommended by unattractive females caused a smaller negative N1 and a greater positive P2. Thus, subjects’ emotions were activated by attractive or unattractive faces transferred to the logo onset; an early attentional emotion valence occurred when the participants viewed the logos, and a more positive emotion was identified when the logos were recommended by attractive females compared with unattractive females. In the later neural process, a more positive LPP was elicited by logos recommended by unattractive females. Previous studies have demonstrated that the LPP was correlated with the emotional processing of subjective evaluation; moreover, negative emotion or a negative human expression would prime a larger LPP compared with a positive emotion and expression^10, 11, 47, 48^. Based on the current findings, one potential explanation of the larger amplitude of the LPP of logos recommended by unattractive female customers may be because when the participants viewed the unattractive female photos, a less positive emotion was triggered, and the kind of emotion transferred and reflected when the logos were presented. Thus, attractive female faces triggered a more positive emotion and were reflected with logo onset. As a result, the argument we proposed was demonstrated: different female facial attraction levels brought different preference degree toward brand logos without significant differences.

There might be another contribute of this study based on the ERP results in experiment 1 (see Fig. 1). We identified three components (N2, P300 and LPP) which were different from some other literatures reported (e.g. N170, P1 and P2) when facial stimuli were presented to subjects^49–51^; thus, we initially proposed an interesting finding of a three-phase neural process when subjects viewed female faces:

### Early automatically perception phase

When the subjects viewed an unattractive female face, an increased N2 amplitude was induced during the 180–280 ms interval. The N2 component has been suggested to be an index of the attention orientation to an emotional stimulus^48^, and an increased N2 has typically been associated with negative stimuli^52–54^. Previous studies on the early perception of attractiveness demonstrated that attractive faces would activate a rapid and automatic perception in a very short time^55–58^. The current findings were different from those of Chen *et al*.^22^ and previous studies; in Chen’s study, attractiveness comprised a variable conducted in a trust game, and a larger negative N2 was elicited by attractive faces. One potential explanation for this difference may be because economic interests were not considered in our experiment. We hypothesized that when economic benefits are not considered, the automatic perception of unattractive female faces may be stronger compared with attractive faces.

### Evaluation phase

An increased positive P300 was identified through the ERP results by attractive female faces. The P300, a late positive component, peaked during 300–600 ms in the central-to-parietal brain area, which has typically been considered a reflection of attentional allocation and motivational salience^35, 59, 60^. Furthermore, P300 has been reported as a valence of reward^61, 62^. In our case, although unattractive faces activate stronger early automatic perception, in the later processing period, attractive faces gained more attention due to a larger amplitude of P300 was observed. It might be because subjects considered seeing these attractive faces as a reward in the task. An increasing body of evidence from functional Magnetic Resonance Imaging (fMRI) has showed that facial attractiveness activated brain areas involved in reward processing, particularly the nucleus accumbens (NAcc), the orbitofrontal cortex (OFC) and ventral striatum^63, 64^.

### Appreciation phase

In this stage, attractive faces continued to make subjects feel more pleasant due to an increased late positive potential (LPP). A greater extent of the LPP component was elicited in response to attractive faces, which was consistent with previous findings^20, 21, 34, 35, 65^. The LPP as a rather sustained positive deflection may comprise evidence in the stimulus-locked event-related potentials following the pleasant onsets compared with neutral images^61^. Johnston and Oliver-Rodriguez^20^ proposed the LPC (late positive component) may represent an affective value interpretation of female facial attractiveness. In our case, attractive female faces (positive stimuli) triggered a larger amplitude of LPP compared to unattractive female faces (neutral stimuli), and it could be a reflection of pleasant emotion was produced when subjects saw attractive female faces. Furthermore, Bamford *et al*.^66^ indicated that LPP congruency effects were positively correlated with behavioural congruency effects, which our results supported (the rating scores of attractive faces were increased compared with unattractive faces). In conclude, female face attractiveness caused the changes in emotion then influenced preference of male subjects.

Although there were no tasks to require subjects to do facial identity or expression processing in our experiments, the three-phase facial evaluation process had also been found and proposed. This finding might be a contribution in recent studies of facial processing. Further, through this cognitive stage, we determined that the emotion triggered by facial stimuli developed preference of female attractiveness and then the preference cross-category transferred to brand logos. And the transfer process impacted subjects’ behaviour and preference attitude. From the perspective of applied economics and management, brand logo does not impact consumers’ behaviour or attitude independently and directly, however, the joint action of a variety of factors produce the brand effect. In our study, we only used facial attraction stimuli as experimental materials, however, we expected that any stimuli developing different preference could cause the same effect we proposed.

Moreover, it was necessary to discuss the differences between preference cross-category transfer effect we proposed and halo effect due to the simulation between them. Halo effect was initially described by Thorndike^67^. In his study, the task was to assess superior officers and other officers in the army and the rating results, which were not exactly the same as the target performed, were higher than expected. One of the branches of halo effect research has been linked to our study, which focused on the attractiveness of humans. The attractiveness halo effect has gradually become a well-documented phenomena in person perception^68^. Individuals of different ages, races and cultures have all demonstrated an attractiveness halo effect^69–72^. Palmer and Peterson^73^ integrated the attractiveness halo effect into perceptions of political expertise and determined that more attractive individuals were assumed to be more persuasive and knowledgeable for political information. Studies have investigated how the halo effect of attractiveness influences the rate of support of congressional candidates and have indicated that attractiveness comprised one predictable factor of vote choices^74, 75^. Wade *et al*.^76, 77^ independently investigated the personality evaluation and perceived life success of women and men with halo effects as a functional weight. The results of their two experiments indicated that halo effects only occurred for social desirability aspects of personality. The main difference between the halo effect and the preference cross-category transfer effect was the preference transfer of the halo effect occurred in the same category (e.g., interpersonal), whereas the effect we proposed occurred across categories accordingly.

## Conclusion

Both the behavioural and ERP data confirmed that cross-category transfer of preference occurred when subjects evaluated their preference degree to a no-name and unfamiliar logo. Brand logos recommended by attractive females generated more positive emotion and increased preference, and the evaluation of female facial attractiveness deeply impacted the valence of other no significantly difference logos. The findings regarding the N1, P2 and LPP components explained the neural process of this “Preference Cross-Category Transfer Effect”. This study demonstrated an old Chinese proverb “” (Love me, love my dog) from a new aspect.

In conclusion, there were two main contributions of this study. First, we may be the first to propose a concept referred to as the “preference cross-category transfer effect” and demonstrated it through neural scientific methods. Second, we proposed a three-phase neural process when male subjects viewed female faces, which included an *early automatic perception phase*, *an evaluation phase and an appreciation phase*. Moreover, this three-phase evaluation process of face attractiveness caused changes in emotion, which subsequently initiated cross-category preference transfer.

Nevertheless, there were several limitations of this study. First, we only included male students as subjects; thus, it remains unclear whether the results would be the same in female subjects. Second, we only considered the impact of positive recommendations; thus, it remains unclear whether the preference degree of the subject would decrease when an attractive female provided negative recommendations. Third, we only used face attractiveness as stimuli in the experiment, in our expectation, other stimuli which could develop different preference would impact subjects’ behaviour and attitude toward logos as well. However, we are unable to investigate the entire suitable stimulus due to the limitation of time and our ability. Fourth, the components we analyzed (N2, P300 and LPP) were kind of different from some literatures studied on face attractiveness as well (such as N170, P1 and P2) and we did not investigated the reasons caused the issue in this study. Further, it needed more evidences and investigations to indicate the differences between preference priming paradigm and affective priming paradigm. Moreover, the three-phase neural process of face evaluation was a preliminary suggestion. It needed to be demonstrated in more details in later studies. These limitations indicated the need for future research.

## Materials and Methods

### Participants

Thirty-eight healthy, right-handed, undergraduate and postgraduate male students participated in this study. They were recruited at Zhejiang University, aged 18–26 years (M = 21.9 years, SD = 1.912 years). All subjects were native Chinese speakers with normal or corrected-to-normal vision, with no history of neurological disorders or mental disease. This study was approved by the Neuromanagement Laboratory Ethics Committee at Zhejiang University. Written informed consent was obtained from all participants prior to the ERP experiment. In experiment 1, the data of two subjects were discarded for misunderstanding the task and excessive recording artefacts, respectively; as a result, thirty-six valid subjects (aged 18–26 years, M = 21.83 years, SD = 1.797 years) were included in the final data analysis, including eighteen subjects for experiment 1 and eighteen subjects for experiment 2.

### Stimulus Material

#### Experiment 1

Eighty individual Chinese female facial images and forty logo images were used in the two experiments. All facial images were collected from Ma and Hu’s^35^ research experimental materials. The faces were not familiar to the participants and did not include singers, TV casts, movie stars or other celebrities. Forty facial images were rated attractive and the other images were rated unattractive according to Ma’s research^34, 35^. Every logo image was contributed by English letters and the same earphones picture only; all images were obtained from the Internet. All the logos were created by the authors and did not exist in real life. The logos were unfamiliar to the subjects and did not include name-brands. Both the facial images and logo pictures were adjusted to a uniform size (4 by 4 cm, 250 by 250 pixels) and grey-processed using Photoshop software to ensure consistency in the background, brightness, contrast, and colour saturation.

Forty logos were primed as earphone brands sold online, and eighty individual females on pictures were primed as customers who bought the earphones and provided anonymous positive recommendations. Forty logos were randomly and equally divided into two groups; one group corresponded to attractive female faces, and the second group corresponded to unattractive faces. The subjects were informed that there were no significant differences in the product appearance, quality, price or functions of the earphones that belonged to each brand.

#### Experiment 2

Female face stimulus was eliminated in this experiment. The subjects directly viewed logos and ranked their preference degree. We also separated the 40 logos into two groups similar to experiment 1. The other steps and program procedures were the same as experiment 1.

### Experimental Procedure

The experimental instructions were provided to the subjects on paper handouts. The participants were seated comfortably in a dim, sound-attenuated and electrically shielded room. The experimental stimuli were presented at the centre of a computer screen at a distance of 100 cm from each subject’s face. A keypad was provided to the participants to select their choices. Prior to the formal experiment, each subject had three practice trials to become familiar with the experimental procedure.

**Figure 4:**
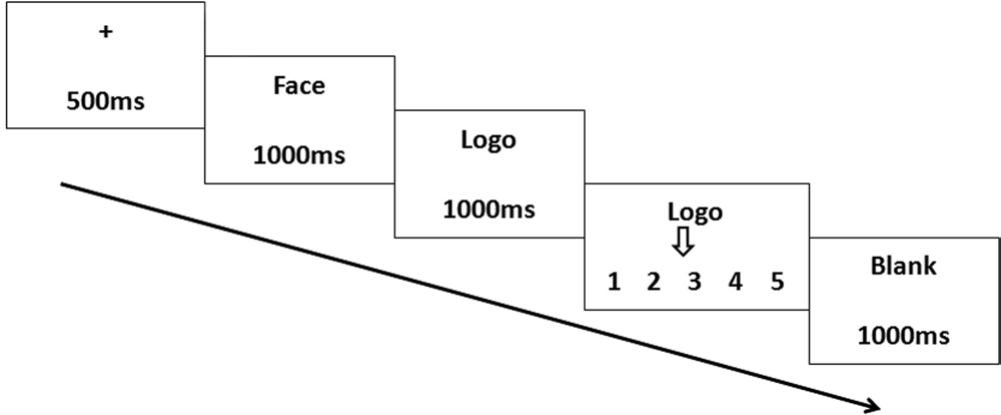
A single trial of the procedure of experiment 1. Participants saw an attractive face or an unattractive face at first then ranked the preference degree of the following logo using a provided keypad.

Figure 4 indicates a single trial in experiment 1. A fixation appeared at the beginning of each trial for 500 ms on the grey screen. An attractive face or an unattractive face was randomly presented for 1000 ms. A logo image was subsequently presented for 1000 ms, and the participants used the number keys 1, 2 and 3 on a mini keypad to rank their preference degree (key 1 and 3 to move the arrow, and key 2 to confirm the result). After the ranking, a 1000 ms duration blank was presented at the end of each trial. There were 80 trials for each participant. Likert scale with 5 points (from 1 = “not attractive at all” to 5 = “extremely attractive) was used as rating method for both facial attractiveness ranking and preference degree ranking of logos. The E-prime 2.0 software package (Psychology Software tools, Pittsburgh, PA, USA) was used for stimuli presentation, triggers and response recording. After the ERP experiment, the subjects were asked to rank the attractive degree of the female facial images.

**Figure 5:**
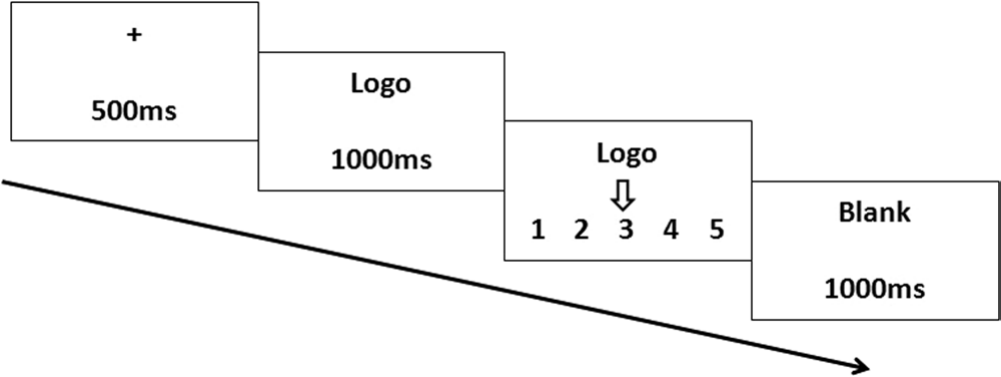
A single trial of the procedure of experiment 2. Participants saw logos directly and ranked the preference degree using a provided keypad.

In experiment 2, the face stimulus was removed, and the subjects ranked their preference degree directly after they viewed the logos. Participants were informed that all the logos were given positive recommendations by consumers before the experiment. There were eighty trials for each participant; each logo was randomly presented two times during the whole testing. The other operation methods were the same as experiment 1 (See Fig. 5).

### Electrophysiological Recordings

EEGs were recorded (band-pass 0.05–70 Hz, sampling rate 500 Hz) with a NeuroScan SynAmps2 Amplifier (Scan 4.3.1, Neurosoft Labs, Inc., Virginia, USA), using a 64-channel electro-cap with Ag/AgCl electrodes, in accordance with the standard international 10–20 system. A cephalic (forehead) location was connected to the ground. The left mastoid was selected as the reference, and the recorded EEGs were off-line re-referenced to the average of the left and right mastoids. For eye movement artefact correction, an electro-oculogram (EOG) was recorded from electrodes placed 10 mm from the lateral canthi of both eyes (horizontal EOG) and above and below the left eye (vertical EOG). The electrode impedance was maintained below 5 kΩ during the experiment.

### Data Analysis

The paired T test statistical method was adopted to analyse the behavioural data. In experiment 1, the comparison of the ranking degree of the logo preference and the attractive degree of the facial images across 2 conditions (attractive and unattractive female faces) was analysed. In experiment 2, we compared the logo preference degrees of the two groups.

EEG data were analysed using the software NeuroScan 4.3.1. The EOG artefacts were initially corrected, followed by digital filtering through a zero phase shift (low pass at 30 Hz, 24 dB/octave). The EEGs were segmented for 1000 ms in each epoch, beginning 200 ms before and continuing until 800 ms after the onset of both the face and logo presentations. The entire epoch was subsequently baseline-corrected using the 200 ms interval prior to the stimulus onset. Trials that contained amplifier clipping, bursts of electromyography activity, or peak-to-peak deflection that exceeded ±80 μV were excluded from the final average. Matlab R2016b was used to generate topographic maps for each condition.

Repeated measures ANOVA was adopted to do the statistics analysis of ERP results according to previous studies^15, 34, 38, 46^. In our study, the dependent variables of ANOVA in experiment 1 with the Face onset were the amplitudes of N2, P300 and LPP, respectively, and the independent variables were face with two levels (attractive vs. unattractive) and electrodes with six levels (F1, Fz, F2, FC1, FCz, FC2). The dependent variables of ANOVA in experiment 1 with the logo onset were the amplitudes of N1, P2 and LPP, respectively, and the independent variables were logo with two groups (logo paired to attractive face vs. logo paired to unattractive face) and electrodes with six levels (F1, Fz, F2, FC1, FCz, FC2). The dependent variables of ANOVA in experiment 2 with the logo onset were the latencies of N1 and P2, and the amplitudes of N1, P2 and LPP, respectively, and the independent variables were logo with two groups (logo paired to attractive face vs. logo paired to unattractive face) and electrodes with six levels (C1, Cz, C2, CP1, CPz and CP2) The degrees of freedom of the F-ratios were corrected with the help of the Greenhouse-Geisser method.

Based on the visual observation and previous studies on facial attractiveness^34, 35, 49^, the peak amplitudes of the central-parietal P300 (in the range of 300–500 ms) and the mean amplitudes of the LPP (from 420 to 580 ms) were mainly analysed to examine the neural process of the attractiveness of female customers. Six electrode sites from central parietal(C1, Cz, C2, CP1, CPz and CP2) were selected for the analysis. Moreover, the early N2 component was also visually observed and analysed. We selected the peak amplitudes from 180 to 280 ms of N2 through six centrofrontal electrode points (F1, Fz, F2, FC1, FCz and FC2). ANOVA factors were stimulus type (two levels: attractive faces or unattractive faces) and electrodes (six levels: F1, Fz, F2, FC1, FCz and FC2 or C1, Cz, C2, CP1, CPz and CP2).

Furthermore, according to the visual observations of the grand average waveforms and topographies (see Figs 2 and 3), we mainly analysed N1, P2 and the central-parietal LPP to examine the neural process of the logo image onset. Six electrodes (F1, Fz, F2, FC1, FCz and FC2) were selected to analyse the N1 component, and the peak amplitude of the 100–180 ms time window was investigated. We analysed the peak amplitude of six electrode points (F1, Fz, F2, FC1, FCz and FC2) from 150–220 ms to observe the P2 component. To observe the LPP component, six electrodes (C1, Cz, C2, CP1, CPz and CP2) were selected, and the ERP amplitude from the time range of 420–550 ms was averaged. Repeated measures ANOVA was also used to investigate the effects across two groups, including one correlated with attractive faces and another correlated with unattractive faces. ANOVA factors were stimulus type (two levels: logos paired to attractive faces or logos paired to unattractive faces) and electrodes (six levels: F1, Fz, F2, FC1, FCz and FC2 or C1, Cz, C2, CP1, CPz and CP2).

Moreover, we analysed N1, P2 and LPP components to examine the neural processes of logo onset in experiment 2. The electrodes and time windows were the same as the logo onset in experiment 1. A repeated measures ANOVA was used to investigate the effects. The ANOVA factors were similar with the factors applied in experiment 1 when analyze the effect of logo onset and the degrees of freedom of the F-ratios were corrected with the help of the Greenhouse-Geisser method either.

### Expermient statement

As corresponding author, Qingguo MA confirmed that all experiments were performed in accordance with relevant guidelines and regulations. All experiments were approved by the Neuromanagement Laboratory Ethics Committee at Zhejiang University, file 001 was a scan copy of the original approval document of the experiments. The red Chinese characters meant that “Reviewed by the ethics committee, this research design respects the personality of the subjects and will not cause psychological and physical damage to the subjects. The experiments are performed in accordance with APA Ethics Code and the principle of International Ethical Guidelines for Biomedical Research Involving Human Subjects by CIOMS.” Further, written informed consent was obtained from all participants prior to the ERP experiments. File 002 showed a sample of informed consent obtained from a subject named “Yaxuan Huang”, additionally, file 003 was a picture of all consent forms signed by all subjects.
